# A novel signature based on microvascular invasion predicts the recurrence of HCC

**DOI:** 10.1186/s12967-020-02432-7

**Published:** 2020-07-06

**Authors:** Binbin Du, Fang Wang, Beers Jarad, Zhihui Wang, Yanzhou Zhang

**Affiliations:** 1 Department of Cardiology, The First Affiliated Hospital of Zhengzhou University, Zhengzhou, 450052 Henan China; 2 Department of Radiology, The First Affiliated Hospital of Zhengzhou University, Zhengzhou, Henan China; 3 Thomas Jefferson University, Philadelphia, PA USA; 4 Department of Liver and Gall, The First Affiliated Hospital of Zhengzhou University, Zhengzhou, Henan China

**Keywords:** Signature hepatocellular carcinoma, mRNA, Microvascular invasion

## Abstract

**Background and objectives:**

In hepatocellular carcinoma (HCC) patients, microvascular invasion (MVI) is associated with worse outcomes regardless of treatment. No single reliable preoperative factor exists to predict MVI. The aim of the work described here was to develop a new MVI− based mRNA biomarker to differentiate between high and low risk patients.

**Methods:**

Using The Cancer Genome Atlas (TCGA) database, we collected data from 315 HCC patients, including mRNA expression and complete clinical data. We generated a seven-mRNA signature to predict patient outcomes. The mRNA signature was validated using the GSE36376 cohort. Finally, we tested the formula in our own 53 HCC patients using qPCR for the seven mRNAs and analyzing the computed tomography (CT) features.

**Results:**

This seven‐mRNA signature significantly correlated with length of recurrence-free survival (RFS) and overall survival (OS) for both the training and validation groups. RFS and OS were briefer in high risk versus low risk patients. A Kaplan–Meier analysis also indicated that survival time was significantly shortened in the high risk group versus the low risk group. Time-dependent receiver operating characteristic analysis demonstrated good predictive performance for the seven-mRNA signature. The mRNA signature also acts as an independent factor according to a Multivariate analysis. Our results are consistent with the seven-mRNA formula risk score.

**Conclusion:**

Our research showed a novel seven-mRNA biomarker based on MVI predicting RFS and OS in HCC patients. This mRNA signature can stratify patients into subgroups based on their risk of recurrence to help guide individualized treatment and precision management in HCC.

## Background

Liver cancer, a highly aggressive form of cancer, is a leading cause of cancer-related deaths worldwide, including China [1]. Hepatocellular carcinoma (HCC) makes up 90% of liver cancer cases. Although developments in medical oncology and surgery have revolutionized the treatment of HCC, the prognosis remains poor, with high recurrence. Recurrence occurs in a quarter of HCC patients after liver transplantation and in more than two-thirds of patients after hepatic resection within 5 years post-remission [2]. Currently, no approved treatment reduces the risk of recurrence, progression, or death. Thus, methods that can predict patient prognosis are urgently needed, so that effective therapeutic and management strategies can be designed for distinct subsets of HCC patients.

The high recurrence of HCC is partly due to microvascular invasion (MVI). MVI is characterized by tumor emboli in vessels, including veins, capillaries, and lymphatic spaces. Tumor cell dissemination is associated with poor outcomes. All the evidence and diagnosis of MVI is post-surgery, based on the histopathology of the specimen. Thus, prior to surgery, very little is known about the diagnosis.

The prognostic scoring systems currently available to predict survival in HCC patients before resection or transplantation involve variables such as the tumor size and number, serum alpha-fetoprotein (AFP), and underlying liver disease [3–6]. These scoring systems make use of clinical information or international criterion, while ignoring the underlying conditions and internal changes. Patients who develop HCC usually have inflammation associated with fibrotic and cirrhotic livers, which is often accompanied by widespread lymphocyte infiltration in patients with chronic hepatitis. Thus, the surrounding tissue microenvironment likely has an important influence on HCC metastatic tendency. These scoring systems may help us understand MVI but the prognostic value of these systems is limited. Other systems incorporate morphology features to the advanced examinations, which is an improvement [7]. These systems also make use of qualified image analysis or Radionics to determine which patients have a higher risk for recurrence. With the advancement of microarray and high-throughput technology, several studies have shown a significant relationship between gene expression profiles and signatures with survival and outcome of cancer patients, including HCC [8, 9]. Several research groups have focused on relating gene expression to imaging. The results of these studies offer more clues for predicting MVI, but are not very clinically applicable. Therefore, more research is needed to improve and highlight the function of biomarkers.

The aim of this study was to establish the differential mRNA expression in MVI + HCC patients compared to MVI− patients. This differential mRNA expression pattern can help physicians make more accurate diagnoses. Better diagnoses can lead to optimization of medical resources, such as limited organ supplies for transplantation, and improved treatment for the individual.

## Materials and methods

### Patients

After approval of the study by the Institutional Ethical Committee of the First Affiliated Hospital of Zhengzhou University, China, 53 patients were enrolled and written informed consent was obtained for all patients. Liver cancer samples were collected from untreated patients between September 2017 and April 2018. A three-phase contrast-enhanced computed tomography examination was conducted in all patients. Under double-blind conditions, two hepatobiliary radiologists with more than 10 years’ experience reviewed and analyzed the CT image features independently. Discrepancies about image features were resolved by consensus review.

### Data research

Level 3 data from The Cancer Genome Atlas (TCGA) for 315 patients with HCC were procured from the Cancer Genomics Browser from University of California Santa Cruz, including mRNA expression profiles and clinical information associated with these samples. Patients who satisfied the following criteria were used for the model development: (a) histologic diagnosis of HCC; (b) available microvascular invasion information, mRNA expression data, and comprehensive clinic pathological and follow-up information; and (c) recurrence-free survival (RFS) between 30 and 3000 days. Publicly available data were collected from TCGA. Thus, further authorization by the institutional ethics committee was unnecessary. GSE36376 was procured from Gene Expression Omnibus (GEO) for the validation group.

### Construction of a risk-score formula

Using the TCGA training set, a univariate Cox proportional regression model comparing MVI+ and MVI− groups differential gene expression was used to identify potential mRNAs. The relationship of these identified mRNAs to RFS was then analyzed. A good method for regression analysis of high-dimensional data, the least absolute shrinkage and selection operator (LASSO) Cox regression algorithm, was implemented for the identification of prognostic genes. In addition, the log2 fold change and average expression levels were utilized for ranking potential candidates. A linear combination of selected genes weighted by their respective coefficients was designed to develop a patient risk score formula. Risk score = (expr Gene1 * coif Gene1) + (expr Gene2 * coif Gene2) + … + (expr Gene5 * coif Gene5).

Identical Ƀ-values were tested in the validation set. Survival curves of the risk scores were generated in the training and validation sets with a Kaplan–Meier survival analysis and a two-tailed log-rank test. Prognostic performance was determined using y calculated from the area under the curve (AUC) from the time-dependent receiver operating characteristic (ROC) analysis. Risk score accuracy for predicting RFS at 1, 3, and 5 years was determined. The independence of the predictive mRNA signature was determined using a multivariable Cox proportional hazards regression analysis, where the dependent variable was RFS and the mRNA signature and other clinical factors were covariables.

### Statistical analysis

R language (Version 3.3.3) was utilized for all statistical analyses. The ‘survminer’, ‘survival’, and ‘survival ROC’ packages were utilized for the creation of survival curves and ROC curves. Differences in clinic pathological features were assessed with the Student’s *t* test or Chi squared test. Statistical tests were two-tailed. P less than 0.05 was considered significant.

## Results

### Determining the prognostic mRNA signature in the discovery group

**Fig. 1:**
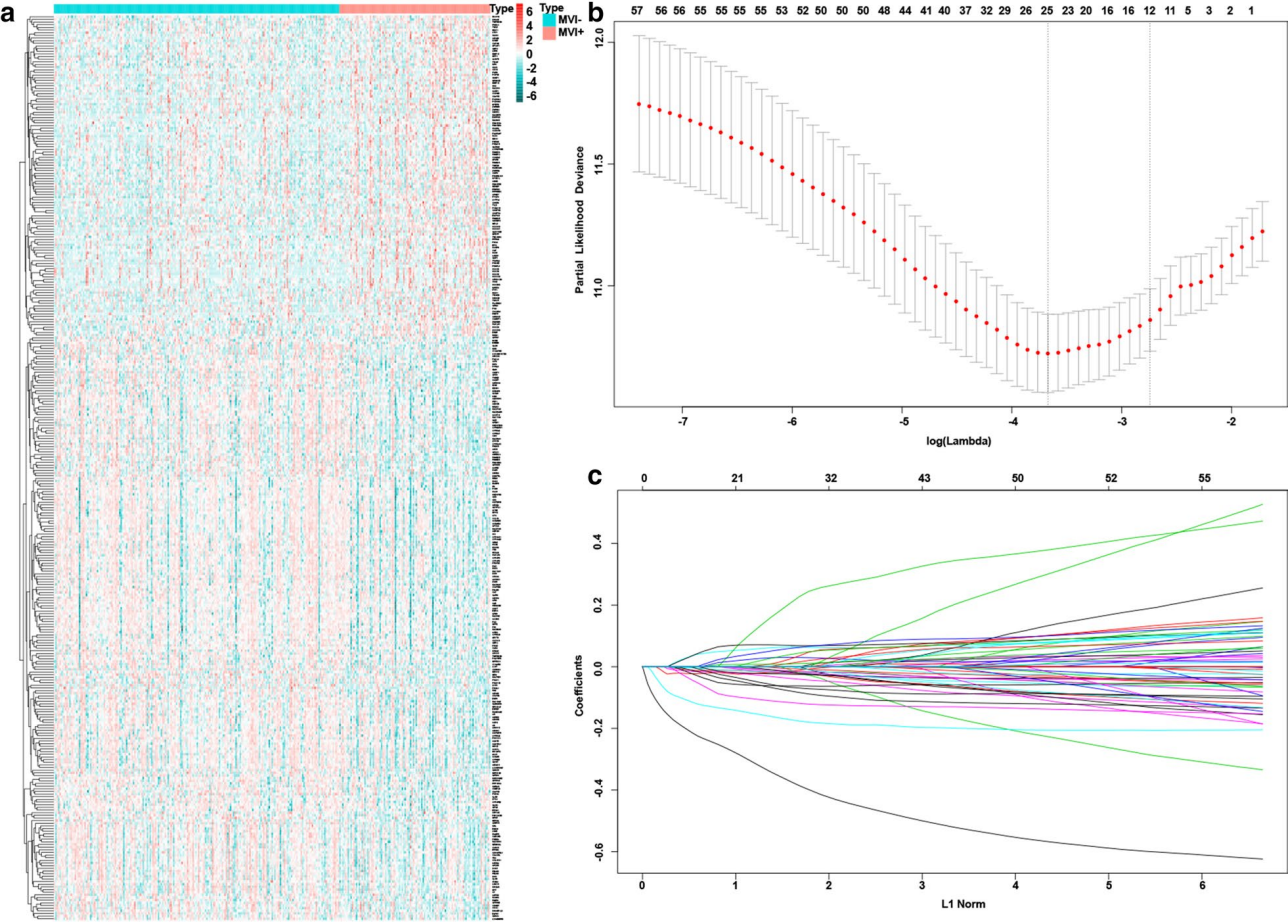
The different mRNA expression between MVI+ and MVI− in TCGA discovery group. **a** Univariate Cox regression analysis shows 341 genes significantly related to RFS. **b** A tenfold cross-validation for tuning parameter selection in the LASSO model. **c** The LASSO coefficient profiles for the associated genes

The model was constructed from the TCGA data of 315 HCC patients. Of the 315 patients, 209 were MVI− and 106 MVI+. The mRNA expression data were compared. The logFC value was > 0.5, P < 0.01. A total of 341 different genes were differentially expressed (Fig. 1a). A univariable Cox proportional hazards regression analysis of the mRNA profiling data from the discovery group was conducted resulting in 59 mRNAs with P-values less than 0.05. A LASSO Cox regression model was utilized to identify the most prognostic genes. We obtained two constitute one is one standard error (SE) of the minimum criteria and minimum criteria [10]. Based on the simplified criteria, the lse model was used (Fig. 1b, c), resulting in 12 candidate genes. Genes were further filtered with a stepwise regression using a multivariable Cox proportional hazards regression model. Seven genes (IM2A, STAG3, ADH1C, NEIL3, GULP1, PPAP2C, CKMT1B) were identified. Correlations among the seven genes in the TCGA cohorts were evaluated using a heatmap. The risk score was calculated using the expression levels of the seven genes and the corresponding Coif. The resulting risk-score formula was: risk score = + (CKMT1B*−0.15788) + (PPAP2C*−0.06074) + (GULP1*0.111848) + (NEIL3*0.114841)) + (ADH1C *−0.10131)) + (STAG3*−0.19945) + (ITM2A*−0.3719).

**Fig. 2:**
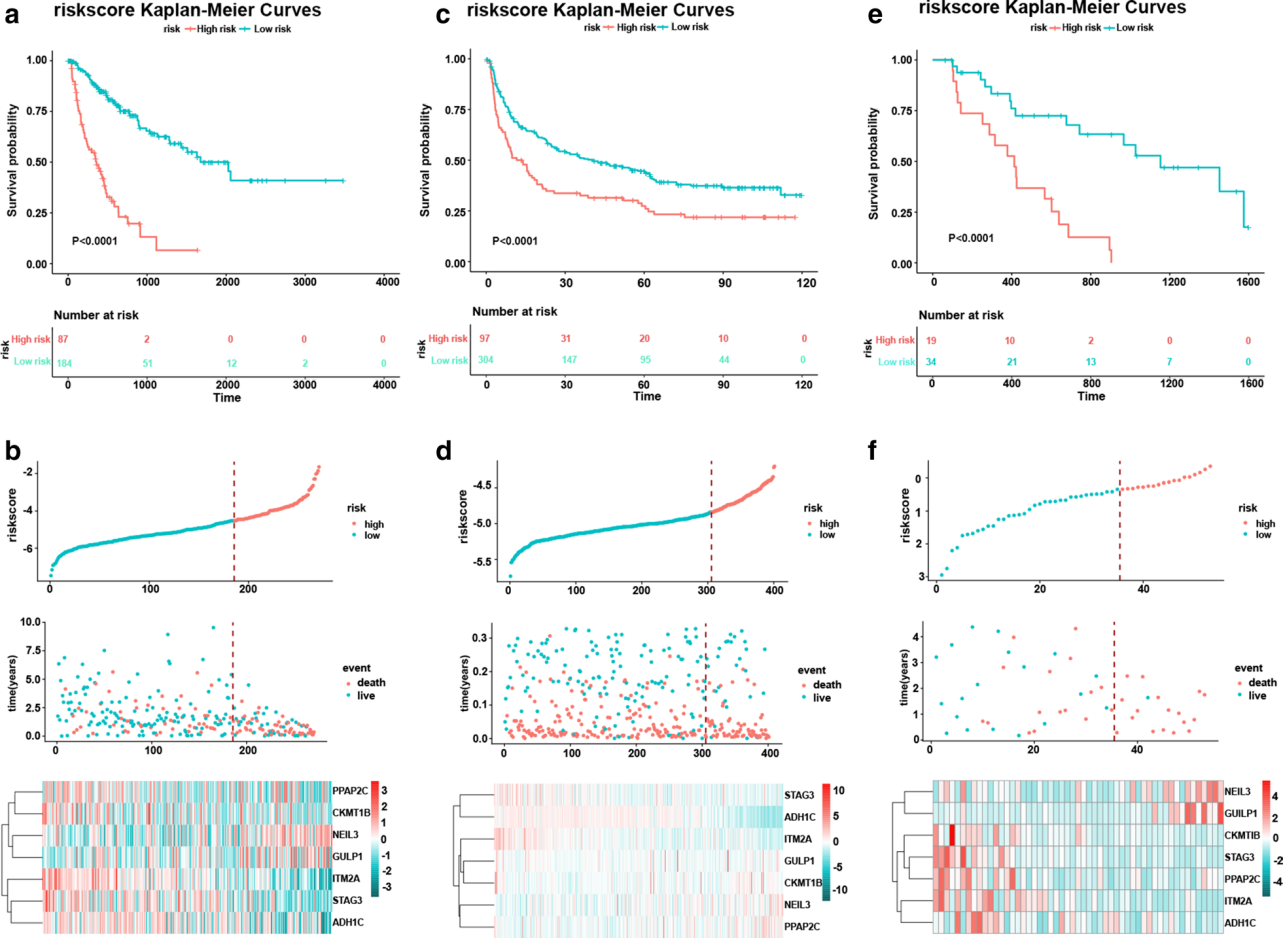
Kaplan–Meier analysis of RFS of the formula in different groups. a Risk formula analysis in the TCGA set: The recurrence time in high and low risk groups were applied using the Kaplan–Meier analysis. **b** The distribution of the risk formula, patients’ status, and the expression heat map for the discovery set. **c** Risk formula analysis in the GEO set: Recurrence time in high and low risk groups based on the Kaplan–Meier analysis. **d** Distribution of the risk formula, patients’ status, and the expression heat map for the discovery set. **e** Risk formula analysis in the 53 patient set: Recurrence time in high and low risk groups based on the Kaplan–Meier analysis. **f** The distribution of the risk formula, patients’ status, and the expression heat map for the discovery set

Patients in the training cohort were categorized as high risk or low risk based on the optimum cutoff(−4.52432). The training cohort consisted of 87 high risk patients with 54 recurrences and 184 low risk patients with 53 recurrences. High risk patients had worse outcomes compared to low risk patients (P < 0.0001; Fig. 2a), as determined by the Kaplan–Meier analysis. Notably, if the mRNA expression value was weighted by a negative coefficient in this formula, the gene was protective with low risk for recurrence; a positive coefficient signified that the gene was harmful to the prognosis. In the discovery group, patients with low risk scores had a longer RFS time (median 572 days) compared to high risk patients (median 239 days). According to the heat map, the protective five mRNAs were increased in the low risk group, while the other two genes (NEIL3, GULP1) were downregulated (Fig. 2b). Thus, the risk-score formula stratified the HCC patients based on the RFS.

The prognostic performance of the seven mRNA signature was assessed with a time-dependent ROC curve analysis. AUC values for the RNA signature were 0.782, 0.793, and 0.749 for predicting recurrence in the TCGA set at 1, 3, and 5 years (Additional file 1: Figure S1). AUC values exceeded 0.7 [11], indicating that our selection mRNA formula was suitable for predicting recurrence in HCC patients.

### Prognostic value of the seven mRNA formula in the validation groups

To validate the seven mRNA risk score formula, we used GSE36376 containing 401 patients. Similar to the discovery group, high risk patients had shorter recurrence times and worse outcomes than low risk patients (P = 0.00024). There were 97 high risk patients with 61 recurrences, whereas there were 304 low risk patients with 184 recurrences. The RFS time in low risk patients was longer compared to high risk patients (Fig. 2c, d). In addition, the five protective mRNAs increased in low risk patients; the opposite occurred in high risk patients. These results are consistent with the data from the discovery group. Thus, these seven mRNAs are risk factors for recurrence in HCC patients, supporting the risk prediction model.

### The relationship between risk scores and clinical pathological factors

We conducted a set of predefined stratified analyses to identify whether the seven mRNAs have an independent predictive ability in the discovery and validation sets separately. A Cox multivariate regression analysis was performed with clinical characteristics, including age, gender, tumor stage, and WHO grade. The RFS probabilities for high and low risk groups, visualized with Kaplan–Meier plots (Additional file 1: Figure S2 and Figure S3), indicate that the mRNA signature is an independent factor for predicting RFS in HCC patients, both in the discovery and validated sets (Table 1).

**Table 1:** Multivariable Cox regression analysis of RFS in HCC patients in the discovery, validation and our patient set

Charcateristic	Multivariable analysis					
	Coef	HR	Lower 95	Upper 95	z	P value
Discovery set (n = 271)						
Risk sore	1.0001	2.7186	2.1975	3.3633	9.2118	*3.21E−20*
Age_ ≥ 65/, < 65	0.2566	1.2925	0.8791	1.9005	1.3048	0.1919
Gender	− 0.0778	0.9251	0.6121	1.398	− 0.3694	0.7118
Neoplasm-histologic-grade	− 0.1161	0.8903	0.6956	1.1394	− 0.9228	0.3561
Pathologic_M	0.066	1.0683	0.863	1.3224	0.6071	0.5437
Pathologic_N	0.1106	1.117	0.911	1.3695	1.0639	0.2873
Pathologic_T	0.0183	1.0185	0.8327	1.2457	0.1785	0.8583
Pathologic_stage	0.0231	1.0234	0.8378	1.2501	0.2268	0.8205
Validation set (n = 401)						
Riskscore	0.96	2.6117	1.5394	4.4308	3.5597	*0.0004*
Age	− 0.1479	0.8625	0.6456	1.1523	− 1.0007	0.317
Gender	− 0.1514	0.8595	0.6312	1.1703	− 0.9615	0.3363
AJCC_T_stage	0.69193	1.9976	1.7208	2.3189	9.0928	*9.66E−20*
Our sets (n = 53)						
Risk.socre	1.4865	4.4217	2.0409	9.5799	3.7685	*0.0002*
TNM.stag1ng	− 0.1199	0.887	0.5506	1.429	− 0.4929	0.6221
Gender	0.6561	1.9273	0.6694	5.5486	1.2161	0.2239
Age	0.0031	1.0031	0.407	2.4722	0.0067	0.9946

### Evaluation of the formula with our 53 patient set

Fifty-three fresh liver cancer tissues were collected from patients (Table 2) without any treatment. Total mRNA was isolated using TRIzol reagent (Invitrogen, Waltham, MA). A PrimeScript RT Reagent Kit was used for reverse transcription of the isolated RNA (Takara Bio, Otsu, Shiga, Japan). Gene expression levels were quantified using qRT-PCR. In the 53 HCC patients, RFS was shorter in the 19 high risk patients versus the 34 low risk patients. In the high risk group, 18 patients had recurrences, while 15 low risk patients had recurrences. The low risk patients had longer RFS than high risk patients (617.5 days VS 413 days). In this patient group, the seven mRNA formula differentiated the high risk from low risk HCC patients, with respect to RFS (Fig. 2e, f).

**Table 2:** Clinical characteristics of patients in the 53 patients set

Demographics	
Gender	
Male	45
Female	8
Age	
Median (range)	50 (28–77)
Risk factors	
HBV+	30
HCV+	8
Alcohol	6
Others	9
TNM stage	
I	15
II	22
IIIA	10
IIIB	5
IIIC	1
MVI+	28
MVI−	25
Tumor features	
Nonsmooth tumor margin	32 + 21−
Irregular circumferential peritumoral enhancement	23 + 30−
TTPVI	34 + 19−
Events	
Recurrence	33
Noreurrence	20
Deaths	
Median follow up(days)	784 (63–1865)
RFS time(days)	454 (63–1597)

TTPVI = two-trait predictor of venous invasion: Internal arteries and hypodense halos

**Fig. 3:**
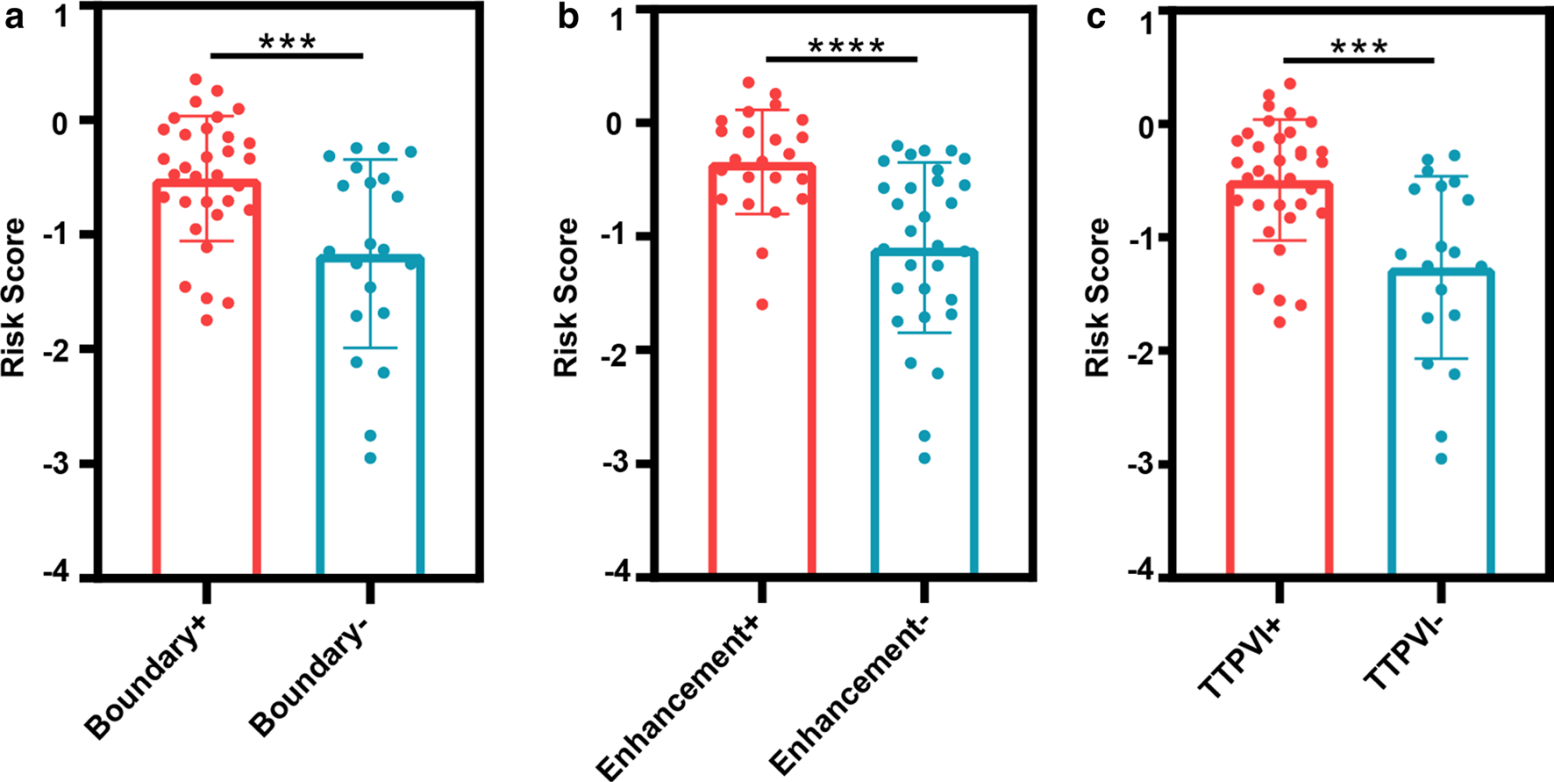
The CT features between the high and low risk group in the 53 patients set: The red column displays the high risk group, while the green column displays the low risk group. **a** The nonsmooth tumor margins, **b** the peritumoral enhancement, and **c** TTPVI

**Fig. 4:**
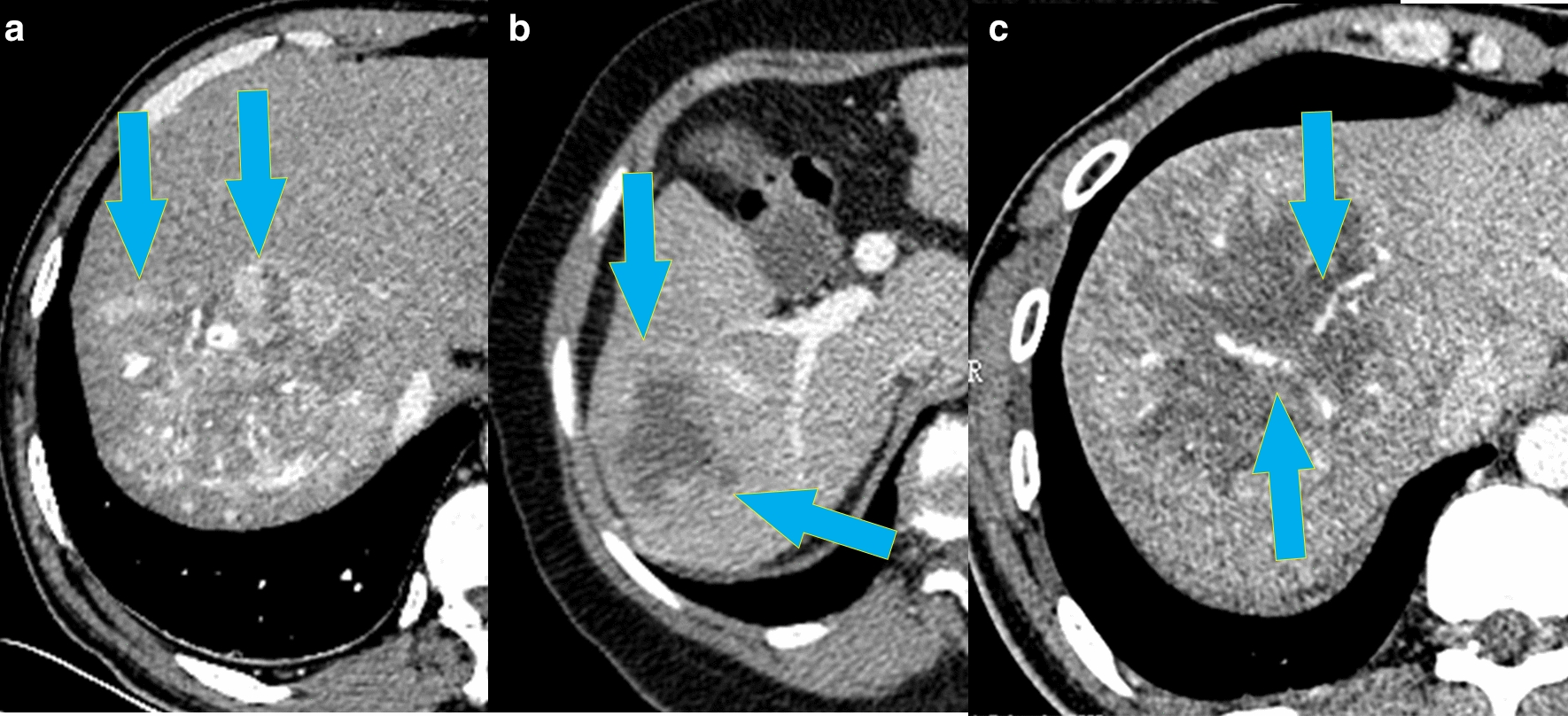
Computed tomography scan revealed the patients with different RFS. **a** peritumoral enhancement: recurrence 3.2 months, external portion enhancing in the arterial phase (arrows). **b** nonsmooth tumor margin positive: recurrence 4.5 months, focal extranodular extension (arrows) in the venous phase. **c** The two-trait algorithm predictive of MVI (TTPVI) positive: recurrence 2.8 months, blue arrows show internal artery and lower image means noncontinuous hypoattenuating halos

Imaging examination is widely used for assessing tumors. Therefore, for our patient group, we evaluated the association between imaging features and the mRNA signature. In previous research, we found that tumor dimension, non-smooth tumor margins, peritumoral enhancement, and TTPVI have the best ability to predict MVI in HCC [12]. Non-smooth tumor margins, peritumoral enhancement, and TTPVI are related to tumor size [13]. Therefore, we did not collect information about tumor size. After comparing the CT images, we found more “worrisome” features in high risk patients versus low risk patients (Fig. 3). Patients had high similarities between the imaging features and the mRNA formula (Fig. 4). In other words, the mRNA formula was consistent with our radiology consensus.

**Fig. 5:**
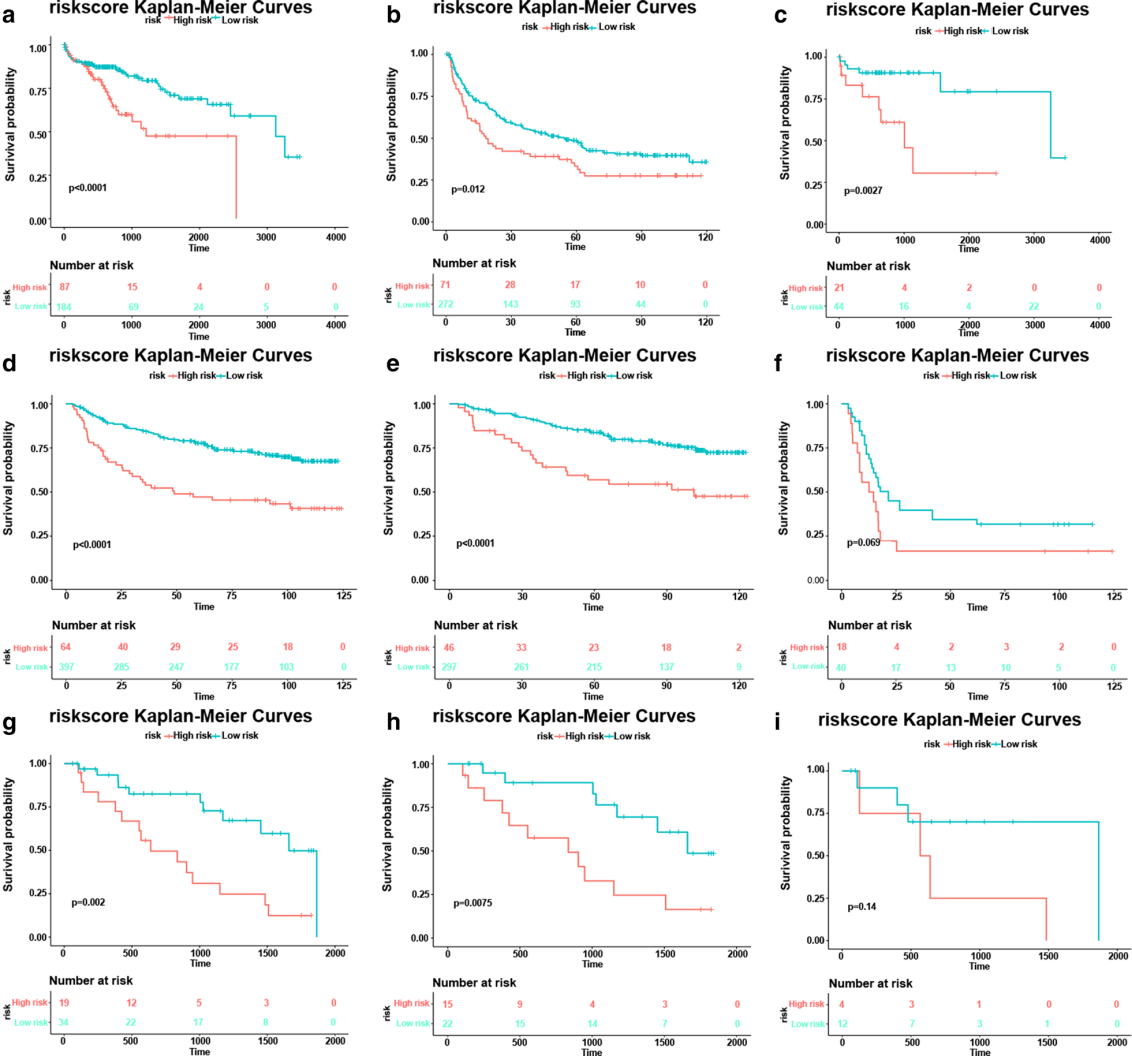
Kaplan–Meier analysis of OS of the formula in different groups and stages. **a** Risk formula analysis in the TCGA set: The OS time in high and low risk groups based on the Kaplan–Meier analysis, **b** Kaplan–Meier curves obtained for TCGA set patient stages I and II. **c** Kaplan–Meier curves obtained for TCGA set patient stages III and IV. **d** Risk formula analysis in the GEO set: The OS time in high and low risk groups based on the Kaplan–Meier analysis. **e** Kaplan–Meier curves obtained for GEO set patient stages I and II. **f** Kaplan–Meier curves obtained for GEO set patient stages III and IV. **g** Risk formula analysis in the 53 patient set: The OS time in high and low risk groups based on the Kaplan-Meier analysis, **h** Kaplan–Meier curves obtained for 53 patient stages I and II, **i** Kaplan–Meier curves obtained for 53 patient stages III and IV

Cancer-related death is the most important index for patients. Thus, we assessed the association of risk score with overall survival (OS) time. Data from the discovery group indicated that patients with low risk scores had longer OS times than patients with high risk scores. Mortality rate in high risk patients was significantly elevated compared to low risk patients (Fig. 5), indicating that the seven-mRNA formula might play an important role in identifying patients with more risk of recurrence and worse outcomes. This formula showed the same effect in our 53 patient set. When patients were stratified according to tumor grade, the seven-mRNA formula could predict the recurrence based on the risks. In other words, the formula has the ability to divide the HCC patients with respect to OS and RFS based on the MVI.

## Discussion

Although diagnosis and management of HCC have advanced, the median patient survival time is less than 8 months; HCC is still a highly malignant cancer [14]. Even if patients choose curative treatment, such as surgical resection, liver transplantation, and local ablation, the frequent recurrence impedes successful outcome. For personalized patient management and elucidation of the biological features of recurrence, the development of a feasible and reliable signature that can predict high risk of recurrence is necessary. In our study, we developed and validated an mRNA formula which predicts recurrence. This is the first research based on mRNA to predict recurrence.

A risk score formula that can estimate HCC recurrence and prognosis has value in guiding the management of HCC. A risk score may help doctors identify candidates for liver transplantation and guide the design of clinical therapies. For example, patients who have high risk scores may not be the best candidates for liver transplantation compared to low risk patients, but may be candidates for more aggressive treatments.

Cancer gene expression can reveal the etiology, prognosis, and treatment response [15, 16]. With gene sequencing advancements, a large number of messenger RNAs (mRNA) have been explored in relation to cancer. Our study revealed that a seven mRNA signature (ITM2A, STAG3, ADH1C, NEIL3, GULP1, PPAP2C, CKMT1B) is associated with HCC recurrence. Patients with high mRNA signature based risk scores have shorter recurrence and OS times. The mRNAs in the predictive seven mRNA risk score shown below were also reported in other cancers. Risk score = + (CKMT1B*−0.15788) +(PPAP2C*−0.06074)) + (GULP1*0.111848) +(NEIL3*0.114841)) + (ADH1C *−0.10131)) + (STAG3*−0.19945) + (ITM2A *−0.3719). CKMT1B is involved in breast cancer. However, in contrast to our study, high CKMT1B expression was associated with poor outcomes [17–20]. CKMT1B encodes for a protein responsible for transferring high energy phosphate from mitochondria to cytosolic creatine. Thus, the upregulated expression of CKMT1B may result in more energy for the growing tumor tissue. This gene is also a porin of the mitochondrial membrane pore and impacts apoptosis. More research is necessary to elucidate the role of CKMT1B in HCC. PPAP2C belongs to the phosphatidic acid phosphatase enzyme family responsible for regulating dephosphorylation of lipid phosphates [21, 22]. This gene is overexpressed in many cancers, including ovarian carcinoma, lung cancer, and bladder, prostate cancers, as well as sarcomas. PPAP2C can regulate cell proliferation and may be an anticancer drug. This is consistent with our finding that this PPAP2C plays a protective role in HCC patients. GULP1 expression is significantly decreased in cancer tissue versus normal tissues and highly hypermethylated. Consistent with our data, GULP1 expression is associated with outcomes in ovarian cancer patients [23, 24]. Elevated expression of NEIL3 occurs in many human cancer cell types and tissues and is associated with primary malignant melanomas and metastasis [25–27]. NEIL3 may be a latent tumor suppressor gene for hepatocellular carcinoma [28]. This is consistent with our risk formula. The ADH1C gene has two alleles (ADH1C*1 and ADH1C*2), which code for the γ1 and γ2 enzyme subunits with different in vitro kinetic properties. ADH1C is associated with susceptibility to oral cancer, and may have anti-oncogenic, proapoptotic effects. In addition, ADHIC may be involved in rapid metabolism of ethanol and accumulation of acetaldehyde in tissue, resulting in increased cancer risk. We found that ADH1C may be protective in HCC. HCC is often associated with alcohol consumption; thus, ADH1C may have the same mechanism in HCC patients [29–31]. STAG3 has a tumor suppression function in ovarian cancer. This is consistent with our finding. STAG3 is also associated with lymphoma, and colorectal and testicular cancers [32, 33]. ITM2A is GATA3-related gene and has been reported as a downstream target in T-cell lymphoma. ITM2A also acts as a tumor suppressor of ovarian cancer via G2/M cell cycle arrest. According to our study, this gene is also protective in HCC [34–36].

This seven-mRNA risk formula was validated in three independent cohorts, including our fifty-three HCC patient set. Because imaging examination is popular in the clinical practice, we also compared our finding and the accepted MVI imaging feature; the results from this comparison are consistent concerning recurrence and OS. All the results showed that this mRNA signature is worthy to apply in clinical practice.

## Conclusion

This seven-mRNA risk formula is an independent factor from other clinical traits for micro-vessel invasion. Therefore, the mRNA risk formula can be a predictor of HCC recurrence. In summary, our finding has tremendous value in the diagnosis and treatment in HCC patients, especially in predicting the recurrence of MVI.

## Supplementary information

**Additional file 1: Figure S1.** Time‐dependent survival ROC analysis of the formula signature in the TCGA set. AUC: area under the curve. **Figure S2.** Supplement. Kaplan-Meier analysis of RFS of the formula in different groups and stages. Risk formula analysis in the TCGA set: The RFS time in high and low risk groups based on the Kaplan-Meier analysis, Kaplan–Meier curves obtained for TCGA set patient stage I (**a**), stage II (**b**), stage III & IV (**c**). Risk formula analysis in the GEO set: The RFS time in high and low risk groups based on the Kaplan-Meier analysis, Kaplan–Meier curves obtained for GEO set patient stages I (**d**), II (**e**), stage III & IV (**f**). **Figure S3.** Kaplan-Meier analysis of RFS of the formula in 53 patients group.Risk formula analysis in the 53 patients group set: The RFS time in high and low risk groups based on the Kaplan-Meier analysis, Kaplan–Meier curves obtained for patient stages I & II (**a**), Kaplan–Meier curves obtained for patient stages III & IV (**b**).

## Data Availability

All data generated or analyzed during this study are included in this article.

## Abbreviations

HCC: Hepatocellular carcinoma
MVI: Microvascular invasion
TCGA: The Cancer Genome Atlas
CT: Computed tomography
RFS: Recurrence-free survival
OS: Overall survival
LASSO: Least absolute shrinkage and selection operator
AUC: Area under the curve
ROC: Time-dependent receiver operating characteristic
TTPVI: Two-trait predictor of venous invasion: Internal arteries and hypodense halos
coif: Coefficient
