# Supplementary material for: A novel signature based on microvascular invasion predicts the recurrence of HCC
Source: J Transl Med. 2020 Jul 6;18:272. doi: 10.1186/s12967-020-02432-7 (PMC7336478; doi:10.1186/s12967-020-02432-7)
Supplement: Supplementary file 1 — Additional file 1: Figure S1. Time‐dependent survival ROC analysis of the formula signature in the TCGA set. AUC: area under the curve. Figure S2. Supplement. Kaplan-Meier analysis of RFS of the formula in different groups and stages. Risk formula analysis in the TCGA set: The RFS time in high and low risk groups based on the Kaplan-Meier analysis, Kaplan–Meier curves obtained for TCGA set patient stage I (a), stage II (b), stage III & IV (c). Risk formula analysis in the GEO set: The RFS time in high and low risk groups based on the Kaplan-Meier analysis, Kaplan–Meier curves obtained for GEO set patient stages I (d), II (e), stage III & IV (f). Figure S3. Kaplan-Meier analysis of RFS of the formula in 53 patients group.Risk formula analysis in the 53 patients group set: The RFS time in high and low risk groups based on the Kaplan-Meier analysis, Kaplan–Meier curves obtained for patient stages I & II (a), Kaplan–Meier curves obtained for patient stages III & IV (b). [file 12967_2020_2432_MOESM1_ESM.docx]

**Supplementary Figures**

**
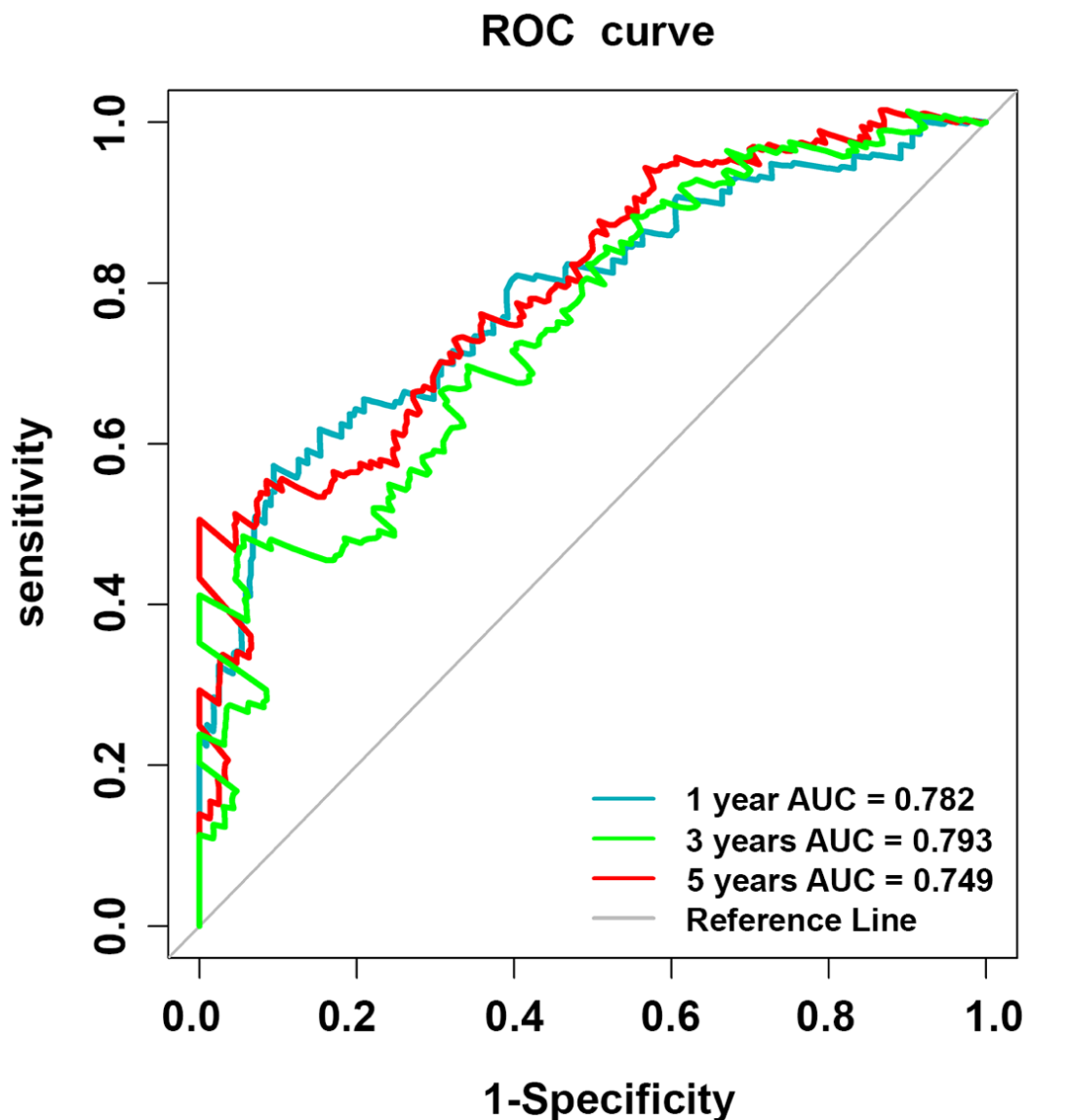
**

Figure 1 supplement. Time‐dependent survival ROC analysis of the formula signature in the TCGA set. AUC: area under the curve.


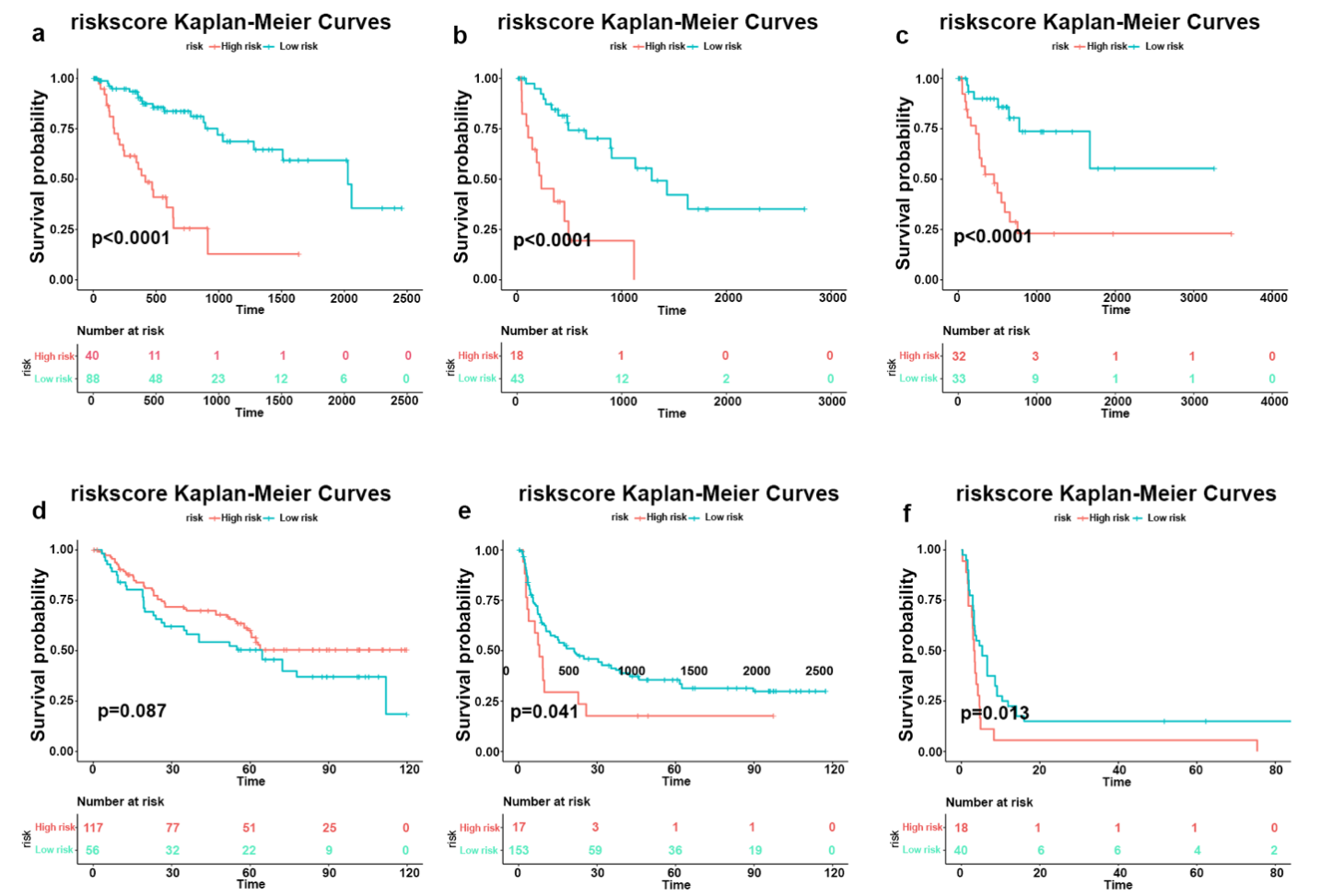


Figure 2 supplement. Kaplan-Meier analysis of RFS of the formula in different groups and stages. Risk formula analysis in the TCGA set: The RFS time in high and low risk groups based on the Kaplan-Meier analysis, Kaplan–Meier curves obtained for TCGA set patient stage I(a), stage II (b), stage III&IV (c). Risk formula analysis in the GEO set: The RFS time in high and low risk groups based on the Kaplan-Meier analysis, Kaplan–Meier curves obtained for GEO set patient stages I (d), II (e), stage III&IV (f).


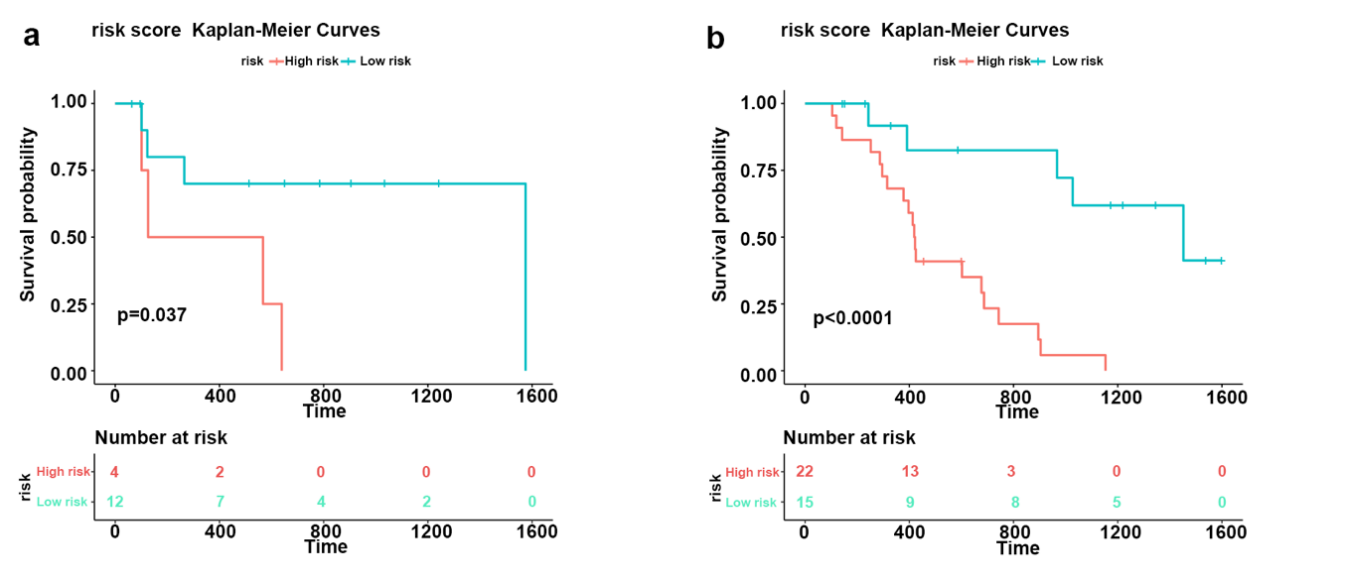


Figure 3 supplement. Kaplan-Meier analysis of RFS of the formula in 53 patients group.Risk formula analysis in the 53 patients group set: The RFS time in high and low risk groups based on the Kaplan-Meier analysis, Kaplan–Meier curves obtained for patient stages I & II (a), Kaplan–Meier curves obtained for patient stages III & IV (b).
